# NEXTODERM: Consensus on Dermatophytosis Diagnosis and Management in Nepal

**DOI:** 10.1155/drp/1347872

**Published:** 2025-10-07

**Authors:** Sudha Agrawal, N. K. Singh, Saraswoti Neupane, Dinesh Binod Pokharel, B. M. Kayastha, D. P. Shrestha, S. L. Rajbhandari, Sanju Babu Shrestha, Sabeena Bhattarai, Dharmendra Karna, Shristi Shrestha, Anupama Karki, Ajay Kumar, Sashi Hirachan, Keshav Dhakal, Kumar Pokhrel, Eliz Aryal, Yogesh Poudel, Sushil Paudel, Rabindra Sharma, Smita Joshi, Niraj Parajuli, Sambhu Datta Joshi, Badri Chapagain, Sudip Parajuli

**Affiliations:** ^1^Department of Dermatology, B.P. Koirala Institute of Health Sciences, Dharan, Nepal; ^2^Department of Dermatology, Skin Care Centre, Kathmandu, Nepal; ^3^Department of Dermatology, Gandaki Medical College, Pokhara, Nepal; ^4^Department of Dermatology, Kathmandu Medical College, Kathmandu, Nepal; ^5^Department of Dermatology, Patan Hospital, Kathmandu, Nepal; ^6^Department of Dermatology, Tribhuvan University Teaching Hospital Maharajganj, Kathmandu, Nepal; ^7^Department of Dermatology, Army Hospital, Kathmandu, Nepal; ^8^Department of Dermatology, Nepal Medicity, Nakkhu, Nepal; ^9^Department of Dermatology, Lavana, Kathmandu, Nepal; ^10^Department of Dermatology, Cutis Care, Kathmandu, Nepal; ^11^Department of Dermatology, Nepal Medical College, Kathmandu, Nepal; ^12^Department of Dermatology, Bir Hospital, Kathmandu, Nepal; ^13^Department of Dermatology, Manipal Teaching Hospital, Pokhara, Nepal; ^14^Department of Dermatology, Bharatpur Hospital, Chitwan, Nepal; ^15^Department of Dermatology, Mechi Hospital, Jhapa, Nepal; ^16^Department of Dermatology, Kohalpur Medical College, Nepalgunj, Nepal; ^17^Department of Dermatology, Butwal Hospital, Kathmandu, Nepal; ^18^Department of Dermatology, Civil Hospital, Kathmandu, Nepal; ^19^Department of Dermatology, DI Skin Hospital and Referral Center, Maharajgunj, Nepal; ^20^Department of Dermatology, Seti Hospital, Dhangadhi, Nepal; ^21^Department of Dermatology, Malampatti, Nepalgunj, Nepal

**Keywords:** consensus, Delphi, dermatophytosis, naïve, Nepal, recalcitrant, tinea

## Abstract

The rising incidence of dermatophytosis, marked by atypical presentations and increasing resistance to treatment, has posed significant challenges to effective clinical management, particularly in regions like Nepal, where localized guidance is limited. In response, a panel of dermatology experts in Nepal conducted a structured literature review and employed a modified Delphi process to develop updated consensus recommendations. These guidelines aim to assist clinicians in making informed decisions regarding diagnosis and treatment, with an emphasis on improving patient outcomes. The consensus highlights key treatment principles, including the potential role of combination therapy and considerations for both localized and more complex presentations of the disease.

## 1. Introduction

Dermatophytosis, a widespread superficial fungal infection, has drawn increased attention in recent years due to its rising incidence. These factors include an increase in incidence, the emergence of more atypical lesions, changes in the mycological profile, and the growing challenge of antifungal resistance [1]. It is classified based on the affected body site, such as tinea corporis (nonhairy skin), tinea barbae (beard), tinea pedis (feet), tinea cruris (groin), tinea capitis (scalp and hair), tinea manuum (hand), and tinea unguium (nails, also known as onychomycosis) [2]. In Nepal and other tropical Asian countries, dermatophytosis primarily affects the 21–30 age group, with a higher prevalence in males (ratio of males to females is 1.39–1) [3]. Contributing risk factors include excessive sweating, advanced age, diabetes mellitus, and certain disorders like lymphomas and Cushing syndrome [4].

Managing dermatophytosis has become increasingly complex due to changing infection patterns and a significant rise in prevalence [5]. However, addressing these evolving challenges is impeded by several treatment-related hurdles. While various antifungal medications like azoles, allylamines, antibiotics, and antimetabolites exist, inadequate treatment responses and the necessity of prolonged treatment durations have become notable concerns. These medications, while effective, also bring forth a host of adverse effects that can further complicate the therapeutic journey [6]. One of the prominent issues contributing to the treatment complexity is noncompliance with therapy. This factor is interlinked with the arduously long treatment periods as shown in one of the Indian studies on real-world scenarios by Dhoot et al. [7], leading to lapses in patient adherence. Further research on dermatophytes is essential, especially in relation to antifungal resistance, clinical and mycological associations, and treatment outcomes. Healthcare is an ever-evolving field, with ongoing research and clinical experience continually enhancing treatment methods. While international guidelines are essential for sharing best practices, they must be periodically updated to incorporate the latest evidence and advancements in the field. This can create a situation where some existing guidelines, while significant contributions in their time may not fully capture the current best practices [8].

A significant global issue is the misuse of topical corticosteroids (TCSs), often available over-the-counter, sometimes combined with antibiotics or antifungals. This misuse has led to adverse dermatological effects, particularly in countries like Nepal, where nonprescription sales and a lack of qualified dermatologists contribute to the problem [9]. The abuse of TCS results in conditions that are difficult to treat and often more severe than the original skin issues. This pattern is causing distressing conditions that are challenging to treat and often more severe than the original skin issues [10].

## 2. Rising Burden of Dermatophytosis in Nepal

The estimated prevalence of dermatophytosis in Nepal is around 4.4%. This infection significantly impacts patients' quality of life, with the highest incidence in the 21–30 age group, slightly more common in males [3]. A survey by Karki et al. found that patients spend a median of NRs. 1500 (10.80 USD) on treatment before seeing a dermatologist. The misuse of over-the-counter topical steroids and poor adherence to prescribed medications lead to treatment-resistant cases. Patients often visit dermatologists late, after using various medications or when lesions become chronic [11]. In Nepal, the availability and abuse of topical drug combinations contribute to chronic, treatment-resistant dermatophytosis. This persistent issue remains a therapeutic challenge for clinicians.

## 3. Caveats in Current Guidelines/Consensus

Research on tinea corporis, tinea cruris, and tinea pedis has been largely neglected. Existing guidelines, such as those by the American Academy of Dermatology, are nearly 2 decades old and may not address current challenges adequately [12]. Recent guidelines from the British Association of Dermatology and the British Medical Journal focus more on tinea unguium and tinea capitis, providing limited information on tinea corporis and tinea cruris. While updated reviews have improved knowledge on topical and oral therapies, there is still a significant lack of well-structured clinical trials and evidence-based guidelines regarding the optimal dosage and duration of systemic antifungal treatments for tinea corporis and tinea cruris [13–16].

## 4. Need for Nepal-Specific Consensus

In 2018, specialists developed a consensus guideline for the management of dermatophytosis, known as the Expert Consensus on the Management of Dermatophytosis in India (ECTODERM India) [8]. First, the consensus focused on the Indian scenario and did not take into account the burden of dermatophytosis in neighboring countries like Nepal. Second, the prevalence of skin diseases differs significantly based on geographic location, seasonal changes, and socioeconomic factors. Nepal's climate ranges from the scorching temperatures of the Terai plains exceeding 45°C to the alpine climate of the northern Himalayan region with temperatures below −30°C [17]. Thus, Nepal experiences diverse climatic conditions, leading to varying patterns of skin diseases across regions. The prevalence of dermatological conditions ranges from 20% in the hilly areas to as high as 62% in the Terai region. Eczemas and pigmentary disorders are most frequently observed in the hills, while fungal infections are the predominant skin condition in the Terai [18]. Third, a seasonal trend is observed for the occurrence of certain skin diseases like fungal infection, acne, and melasma [19]. Navigating the varied challenges centered around dermatophytosis necessitates customized strategies to meet the specific needs of each area.

## 5. Materials and Methods

As this process did not involve experiments with human participants or animals, ethics committee approval was not taken.

**Figure 1:**
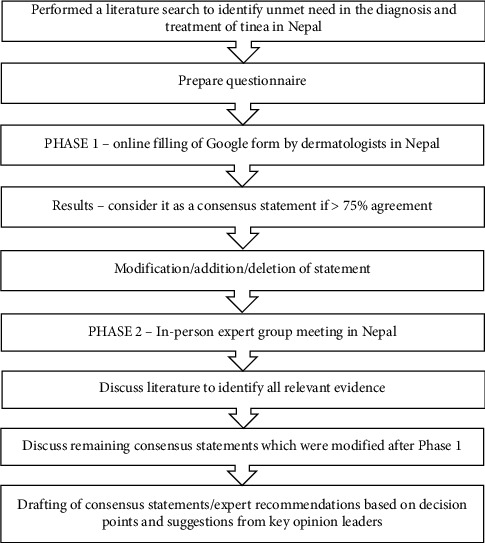
Methodology of the consensus.

The Nepal Expert Forum Consensus Group was established to address the lack of research and updated guidelines on tinea infections in Nepal. The group's goal was to provide updated recommendations to help clinicians choose appropriate treatments for optimal patient outcomes. A team of 25 clinical dermatology experts conducted a literature review to draft consensus statements using a modified Delphi technique (Figure 1).

In Phase 1 (May 2023), a questionnaire with 75 questions on epidemiology, diagnosis, and current management of tinea infections was sent to 25 dermatologists and a mycologist via a Google form. A concordance rate threshold of over 75% was established to achieve consensus for each question, and the consensus statements were formulated based on the collected responses.

Phase 2 involved resharing the questionnaire in a physical meeting in June 2023, where structured discussions covered aspects like current burden, diagnosis, management practices, choice of antifungals, dosage regimens, and combination therapy. The meeting was moderated by a senior consultant dermatologist from Nepal. Consensus statements were developed based on scientific evidence, experience, and collective clinical judgment. The final document was carefully reviewed and revised to include input from all participants. The process followed to develop the consensus statements is illustrated in Figure 1.

The resulting document went through a comprehensive review and was revised to include the feedback and suggestions provided by all participants.

## 6. Expert Opinion/Panel Discussion

### 6.1. Epidemiology

Evidence: In Nepal, dermatophytosis shows a higher prevalence among individuals aged 21–30 years (29.1%) and 31–40 years (21.8%), with both males and females being most commonly affected in the 21–30 age group [3]. This pattern is consistent with findings from Ameen et al. which also identified the 21–30 age group as the most affected, with a prevalence of 31.33% [20]. These results suggest that increased physical activity and higher exposure to the infection contribute to the heightened incidence in this age range.

Consensus: Dermatophytosis is slightly more prevalent in men and commonly affects individuals aged 25–35 years. In clinical practice, over 50% of patients suffer from recalcitrant dermatophytosis, encountering approximately 10–30 cases of dermatophytosis weekly in Nepal. Contributing factors include prior use of TCS, low-quality antifungal drugs, and poor medication adherence.

The guidelines from ECTODERM India and IADVL Task Force against Recalcitrant Tinea (INTACT) are widely preferred, with deviations being uncommon. Reasons for deviation typically include patient-specific factors, drug availability, and cost considerations. These guidelines are pivotal in managing glabrous tinea effectively, ensuring standardized approaches to treatment.  Table 1 outlines the epidemiology of dermatophytosis.

### 6.2. Definitions

Consensus: Table 2 outlines definitions of various terminologies.

### 6.3. Diagnosis

Evidence: Accurate diagnosis guides effective treatment, blending clinical assessment with traditional techniques like KOH microscopy and fungal culture to evaluate fungal morphology and physiology. The complex interplay of demographic trends, clinical manifestations, genetic predispositions, and diagnostic methodologies underscores dermatophytosis's multifaceted nature. In Nepal, patient history and clinical assessment are gold standards, while molecular methods are mainly used in research [21].

#### 6.3.1. KOH Microscopy

The KOH mount is a quick, cost-effective, and simple diagnostic test to perform, requiring minimal infrastructure but some experience to interpret. However, it is not highly specific for onychomycosis or dermatophytosis and cannot monitor treatment. There is a risk of infection spread and potential patient discomfort. While useful in dermatology for diagnostic information, KOH microscopy and fungal culture do not provide early or specific diagnoses [22]. However, conventional methods like KOH microscopy and fungal culture are limited in providing an early and specific diagnosis.

#### 6.3.2. Fungal Culture

According to the American Family Physician, tinea capitis requires treatment with systemic antifungal medications since topical treatments are unable to reach and penetrate the hair shaft effectively [23]. Dextrose agar serves as a versatile medium used for the cultivation of various types of fungi. Commercial culture media for research are costly, necessitating cheaper alternatives [24]. Though fungal culture provides definitive fungal identification, it lacks sensitivity, has a prolonged turnaround time, and is not widely available [8]. Mycologic culture is seldom necessary for diagnosing tineas other than tinea unguium and tinea capitis, and its routine use is generally avoided [25].

#### 6.3.3. Other Advice

Antifungal resistance in dermatophytes could be due to several mechanisms, such as activation of signaling pathways to antifungal stress response, modification in the target site, increased drug efflux, and decreased drug uptake. Some other antifungal resistance mechanisms are efflux pumps, environmental stress, and mutations in the squalene epoxidase gene, which mainly contributes to allylamine resistance, biofilm formation, and antifungal resistance [26].

Consensus: The expert panel unanimously recommended a 10% KOH mount as the point-of-care test for confirming dermatophytosis. Although fungal culture is considered the gold standard for diagnosis, it should be transported in black paper to prevent contamination. However, its routine use in clinical settings is discouraged and instead recommended for stubborn or widespread cases, such as multisite tinea involving tinea cruris and tinea corporis.

The panel also explored the use of dermoscopy as a supportive tool in the diagnosis and management of dermatophytosis, while noting the limited evidence supporting some of its applications. Specifically, the involvement of vellus hairs, nonpigmented hairs lacking medulla or arrector pili muscles has been proposed as an indicator for systemic therapy in select cases. However, published data on vellus hair involvement in dermatophytosis remain scarce, and it is more commonly reported in zoophilic or ectothrix infections. As such, while dermoscopy may aid in identifying features suggestive of deeper or refractory infection, its use as a screening tool for vellus hair involvement across all presentations should be approached with caution. Systemic therapy may be considered in cases where vellus hair infection is confirmed and topical treatments prove inadequate. The mycologist's opinions were largely in agreement with the dermatologists, with a few differences. The mycologist recommended KOH microscopy in over 50% of cases, particularly in suspected, doubtful, naïve, recurrent, chronic infections, steroid-modified cases, treatment failures, and for research purposes. They also recommended fungal culture in more than half of the cases, using clean white paper for transportation. Proper collection and transportation of specimens are crucial for accurate diagnosis. The mycologist attributed antifungal resistance to mutations affecting drug-target genes and emphasized the value of dermoscopy in managing dermatophytosis.  Table 3 outlines the diagnosis of dermatophytosis.

### 6.4. Management Strategy

#### 6.4.1. Tinea Infection

Evidence: Clinical trials have demonstrated fluconazole's efficacy in treating dermatophytosis, though concerns exist due to high minimum inhibitory concentrations observed in recent studies [13]. Conversely, itraconazole is recognized as highly effective, with in vitro data supporting its use and rare resistance occurrences [26]. Studies indicate that itraconazole is the most potent oral azole against dermatophytes [27, 28]. Topical azoles, including luliconazole, sertaconazole, efinaconazole, and lanoconazole, consistently show strong in vitro activity with low minimum inhibitory doses. However, caution is needed with increased azole exposure to prevent resistance development over time.

The initial case of primary resistance to terbinafine in *Trichophyton rubrum* was associated with a mutation in the squalene epoxidase gene, leading to treatment failure [29]. While earlier reports suggested that terbinafine resistance was rare, more recent studies have indicated otherwise—particularly with the emergence of *Trichophyton indotineae*, which has been associated with widespread terbinafine resistance. This is especially relevant in regions like Nepal, where *T. indotineae* infections are prevalent. A recent U.S.-based study reported terbinafine resistance in over 18% of dermatophyte isolates, highlighting the need to reconsider earlier assumptions about its rarity [30]. This evolving resistance pattern underscores the complexity of antifungal stewardship and the need for updated diagnostic and treatment strategies in dermatophytosis management [26].

Consensus: The preferred approach in managing patients with tinea infection involves combination therapy, particularly for extensive or persistent cases. Among topical antifungal agents, luliconazole and butenafine were identified as preferred options by the panel. While scientific data suggest these agents have comparable efficacy to other antifungals within their respective classes, the panel noted that in the local context of Nepal, factors such as cost-effectiveness, availability, and patient adherence influenced their recommendation. These agents are more affordable in Nepal, making them a practical first-line choice. Following lesion clearance, the continued application of topical antifungals for an additional 2 weeks is advised, covering an area extending 2 cm beyond the lesion's borders. In cases with coexisting secondary infections, a short course of oral antibiotics may be considered. When systemic antifungal therapy is used, continuation for 2 weeks postlesion clearance is recommended to reduce the recurrence risk.  Table 4 outlines the management of tinea infection.

#### 6.4.2. Effectiveness of Combination Therapy in the Management of Recalcitrant Tinea Infections

Evidence: According to Cochrane's findings, chronic tinea pedis or cases where topical treatments are ineffective often require systemic therapy. For recalcitrant cases, most dermatologists prescribe topical and systemic antifungal therapy for 8–12 weeks [31]. In a real-world evidence study, patients with recalcitrant tinea had taken oral antifungal for 2–8 weeks [32]. In a previous survey-based study, a total of 38.9% of dermatologists concurred that managing chronic dermatophytosis requires an extended course of itraconazole (6–8 weeks) along with a higher dosage of 200 mg twice daily [33].

Consensus: The systemic and topical antifungal combination is especially favored in cases of extensive lesions or recalcitrant infection. The most preferred therapeutic strategy involves the administration of oral itraconazole alongside topical azoles. However, topical antifungal agents, including Whitfield ointment, are not recommended for recalcitrant tinea infections.  Table 5 outlines combination therapy in the management of recalcitrant tinea infection.

#### 6.4.3. Duration of Treatment

Evidence: As per the recommendation by the American Academy of Family Physicians, topical antifungals should be continued for at least 1 week postclinical resolution [34].

In treatment-naïve cases, topical antifungals are typically applied once or twice daily for a duration of 2–4 weeks. Interdigital tinea pedis may respond to just 1 week of therapy. However, in cases of recalcitrant tinea, topical treatment usually extends beyond 4 weeks. The selection of therapy often depends on regional practices and the clinician's personal experience [13].

Duration of systemic antifungal: itraconazole.

In a comparative study, patients with tinea cruris and tinea corporis treated with itraconazole for 4 weeks achieved effective mycological cure. The treatment led to a marked reduction in pruritus, scaling, and erythema between weeks 0 and 4 [35]. Another study reported that patients with dermatophytosis experienced higher rates of both clinical and mycological cure within a 6-week period [36].

Consensus: According to expert consensus, the optimal treatment duration for topical therapy in patients with localized or initial tinea infections is 4–6 weeks. After achieving a clinical cure, it is recommended to continue topical treatment for an additional 2 weeks. When it comes to systemic therapy for localized or initial tinea infections, the use of intermittent oral itraconazole pulse therapy (200 mg bid for 7 days) is not preferred. This approach involves taking itraconazole in pulses with a 3-week interval between each pulse. Experts have cautioned against using this regimen for localized infections. Interestingly, the experts unanimously agreed that medications administered for shorter durations at higher doses have a lower likelihood of promoting resistance compared with using lower doses for an extended period.  Table 6 outlines the duration for the management of dermatophytosis.

#### 6.4.4. Dosing

Evidence: Table 7 outlines the therapeutic regimens for antifungal medications.

Consensus: As per expert recommendations, for localized or initial tinea infections, the preferred antifungal agents of choice are either Itraconazole (100 mg twice daily) or terbinafine (250 mg once daily). Griseofulvin is not a preferred option for localized infections. In recalcitrant tinea infections, terbinafine (250 mg twice daily) is the recommended choice. It is crucial to note that topical steroids are strictly contraindicated in the management of tinea infections.  Table 8 outlines dosing for the management of dermatophytosis.

#### 6.4.5. Use of Corticosteroids

Corticosteroids, regardless of the form, should be used cautiously as they result in atypical clinical presentations and complicate diagnosis and contribute to treatment failure. Some patients may need steroids—the presence of inflammation may necessitate the use of a combination antifungal/steroid agent. However, steroids must be administered carefully due to the risk of skin atrophy and other steroid-related side effects. The combination of the steroid component offers quick relief from symptoms, and the antifungal agent, although slower acting, works to eliminate the causative organism [33].

Patient profile for prescribing steroids is as follows.

It is advisable to utilize a steroid/antifungal preparation exclusively after confirming a fungal infection diagnosis rather than when its presence is uncertain. Long-term steroid use may cause atrophy, steroid-induced acne, rosacea, and striae [25].

#### 6.4.6. Management of Different Types of Tinea Infection

Tinea capitis requires systemic antifungal treatment, as topical medications are unable to penetrate the hair shaft effectively. In the case of tinea pedis, complications such as secondary bacterial infections can arise—interdigital maceration, skin fissures, and peeling of the stratum corneum can act as entry points for bacteria, potentially necessitating supplementary antibiotic treatment [23].

#### 6.4.7. Management [8].

Tinea pedis is primarily managed with topical treatments, with solutions, gels, or sprays being the preferred formulations. For cases involving dry, scaly, and hyperkeratotic lesions, creams or ointments are more suitable. It is important to use broad-spectrum antifungal agents that are effective against dermatophytes, yeasts, and molds. Imidazoles are often favored by experts due to their dual action—providing strong antifungal activity along with effective antibacterial properties, particularly against Gram-positive bacteria. Other effective topical options for tinea pedis include allylamines, ciclopirox olamine, and amorolfine.

Consensus: According to expert opinions, tinea pedis can act as a reservoir for dermatophyte infections in other parts of the body. For managing recalcitrant lesions and extensive cases, a combination of topical and systemic antifungal treatments is preferred. In the treatment of tinea capitis, both topical and systemic antifungal are used as first-line therapies. Griseofulvin and itraconazole are the preferred systemic antifungal agents for tinea capitis. Systemic antifungal treatment typically lasts for 6–8 weeks in this scenario. Itraconazole is the first-line treatment for onychomycosis caused by dermatophytes, typically administered at a dose of 200 mg daily for 12–16 weeks. This can be given either as continuous therapy or as “pulse therapy” with a dosage of 400 mg per day.  Table 9 presents the management strategies for different forms of tinea infections.

#### 6.4.8. Supplemental Therapy

##### 6.4.8.1. Topical Salicylic Acid as Monotherapy in Recalcitrant Cases

According to Saoji et al., salicylic acid peel is a cost-effective option for treating dermatophytic infections [37]. Salicylic acid is more effective as an adjunctive treatment rather than as a standalone therapy. Salicylic acid 30% acts as a superficial chemical peeling agent, penetrating only the stratum corneum or granulosum. Dermatophyte spores can persist within hair follicles, including vellus hairs, which play a role in the lack of response to standard treatments. Unqualified practitioners using salicylic acid peeling for dermatophytosis could lead to detrimental consequences [38].

##### 6.4.8.2. Off-Label Use of Calcineurin Inhibitors

Topical calcineurin inhibitors (TCIs), originally approved for atopic dermatitis, have also shown effectiveness in managing conditions such as seborrheic dermatitis, genital lichen sclerosus, oral lichen planus, psoriasis affecting the face and flexural areas, vitiligo, and alopecia areata [39]. In cases of dermatophytosis, TCIs work synergistically with ergosterol biosynthesis inhibitors to combat dermatophytes. In addition, topical tacrolimus can enhance compliance with antifungal treatment in recalcitrant of tinea incognito [40].

##### 6.4.8.3. Isotretinoin

The addition of isotretinoin to terbinafine offers no added benefit in treating recurrent dermatophytosis. Isotretinoin's rapid cell turnover may deplete terbinafine's reservoir effect. Isotretinoin reduces sebum production, potentially decreasing the concentration of terbinafine delivered by sebum [41].

##### 6.4.8.4. Isotretinoin as an Adjuvant

The combination of low-dose isotretinoin with itraconazole has shown to be a safe, effective, and promising approach for treating chronic recurrent dermatophytosis, leading to faster complete cure and a marked reduction in recurrence rates. Adequate studies on the pharmacokinetics of itraconazole, with and without isotretinoin, should be conducted to determine differences in drug levels in the stratum corneum and sebum [42].

##### 6.4.8.5. Isotretinoin + Itraconazole

Comprehensive studies are needed to evaluate the impact of isotretinoin on the pharmacokinetics of itraconazole, specifically assessing differences in drug levels within the stratum corneum and sebum, both with and without the coadministration of isotretinoin [43].

Consensus: The consensus among experts is that skin hygiene plays a crucial role in the treatment of dermatophytosis. In addition, oral antihistaminic agents are recommended for managing recalcitrant tinea infections. Emollients and moisturizers are also used in the treatment of tinea infections. However, topical salicylic acid 6% is not considered appropriate for managing recalcitrant tinea infections, and the same applies to TCIs. Furthermore, oral isotretinoin is not recommended for the management of recalcitrant tinea infections.  Table 10 outlines management of various types of supplemental management of tinea infection.

## 7. Consensus on Current Challenges and Unmet Needs

Table 11 provides an overview of the statements that failed to achieve consensus, along with the reasons behind this outcome.

## 8. Clinical Protocol/Algorithm

**Figure 2:**
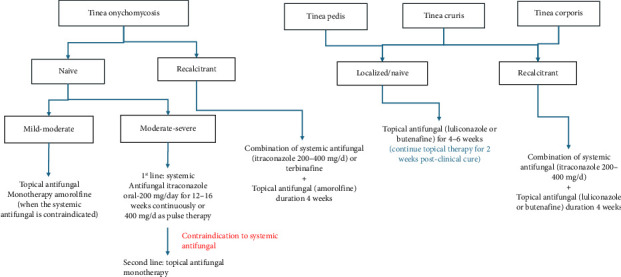
Management of Tinea infections ^∗^Mild–moderate tinea onychomycosis: Superficial fungal infection of the nails, 20%–65% involvement of the nail plate; moderate–severe tinea onychomycosis: Advanced fungal infection of the nails with a higher degree of severity.

Figure 2 illustrates the comprehensive management strategies employed for tinea infections, integrating both systemic and topical antifungal therapies tailored to specific clinical presentations and treatment responses.

## 9. Conclusion

Dermatophytosis is a significant health concern in Nepal, often worsened by the overuse of steroid-based prescriptions. The NEXTODERM consensus aims to bridge research gaps and offers a unified approach to tackle dermatophytosis. This consensus benefits patients by providing improved diagnosis, appropriate treatment options, and a better understanding of the disease. Healthcare professionals gain access to evidence-based recommendations tailored to Nepal's context, leading to better patient outcomes and reduced inappropriate steroid use. NEXTODERM addresses research gaps and provides a relevant framework for healthcare providers, ensuring the best care for patients, promoting awareness, discouraging steroid overuse, and focusing on innovative research and collaboration. Future research should prioritize epidemiological studies, development of cost-effective diagnostic tools, continuous surveillance of antifungal resistance, assessment of public awareness campaigns, and enhancement of healthcare facilities' capacity to diagnose and manage dermatophytosis.

## Figures and Tables

**Table 1:** Consensus on the epidemiology of dermatophytosis.

#	Parameter	Consensus	% agreement
1	Proportion of male and female genders affected by dermatophytosis	60:40 proportion of male: female genders are affected by dermatophytosis	91.30%
2	Age group	Dermatophytosis is commonly seen in 25–35 years of age group	95.65%
3	Reasons for increasing prevalence of recalcitrant dermatophytosis	Prior TCS use	78.26%

**Table 2:** Consensus on definitions of dermatophytosis.

#	Parameter	Consensus	% agreement
*Definitions*			
	Naïve infection	The individual has no prior history of dermatophytosis or its treatment	96.2%
	Chronic dermatophytosis	Dermatophytosis is classified as chronic when it persists for over 6 months to a year, with or without recurrence, despite appropriate treatment	96.2%
	Recurrent dermatophytosis	Re-appearance of dermatophytosis within few weeks (< 6 weeks) of completing treatment is considered a recurrence of the disease	84.6%
	Relapse	A relapse occurs when lesions of dermatophytosis return after the patient has remained symptom-free for 6–8 weeks after successful treatment	84.6%
	Recalcitrant dermatophytosis	Persistent glabrous tinea typically occurs in scenarios such as chronic, recurrent, corticosteroid-altered, or treatment-resistant cases, where there is minimal or no improvement with standard therapies	96.2%

**Table 3:** Consensus on the diagnosis of dermatophytosis.

#	Parameter	Consensus	% agreement
*KOH microscopy*			
	Clinical recommendation	KOH microscopy is recommended in patients with dermatophytosis	88.5%
	% of cases advised for KOH microscopy	KOH microscopy is recommended in less than 10% of the cases	77.78%
	Patient profiles	Doubtful cases	79.17%

*Fungal culture*			
	% of cases advised for fungal culture	Fungal culture is recommended in less than 10% of dermatophytosis cases	80%

*Other advice*			
	Transportation of specimen	Black paper is used to transport the specimen	80%
	Use of sensitivity pattern	Knowing the sensitivity pattern of dermatophytes from chronic/recurrent/recalcitrant tinea cases will help to develop optimum treatment guidelines	96.2%
	Dermatoscope	Dermatoscope helps in the management of dermatophytosis	95%

*Other investigations*			
	LFTs/RFTs before treatment initiation	LFTs/RFTs are NOT to be advised at the beginning of systemic antifungal treatment	85%
	LFTs/RFTs for toxicity monitoring	LFT and RFT should be monitored if any systemic antifungal is advised to the patients	84.6%

*Note:* KOH, potassium hydroxide.

Abbreviations: LFTs, liver function tests; RFTs, renal function tests.

**Table 4:** Consensus on the management of tinea infection.

#	Parameter	Consensus	% agreement
*Management of tinea infection*			
	Treatment strategy	Combination therapy is the most widely recommended approach for managing patients with tinea infections	96.7%
	Commonly used topical antifungal in tinea infection	Luliconazole and butenafine are the top-choice topical antifungals commonly used in the treatment of tinea infections.	84.6%
	Duration of topical antifungal	Topical antifungal agents are recommended for another 2 weeks after the lesions of tinea infection have resolved	93.5%
		Topical antifungal agents are recommended to be applied extending 2 cm beyond the edges of the lesion	93.5%
		Use a course of oral antibiotics if secondary infection coexists	83.9%
	Duration of systemic antifungal	Systemic antifungal agents are recommended for another 2 weeks after clearance of lesions of tinea infection	80.95%

**Table 5:** Consensus on combination therapy in the management of recalcitrant tinea infection.

#	Parameter	Consensus	% agreement
	Type of combination therapy	The use of both systemic and topical antifungal agents in combination is a commonly favored approach for treating tinea infections	93.5%
	Patient profile	Patients with extensive or persistent tinea infections are often treated with a combination of systemic and topical antifungal medications	75%
	Most preferred systemic and topical antifungal combination	Oral itraconazole plus topical azoles	75%
	Whitfield ointment	Topical antifungal and Whitfield ointment are NOT used for recalcitrant tinea	76.19%

**Table 6:** Consensus on the duration for the management of dermatophytosis.

#	Parameter	Consensus	% agreement
*Localized and naïve tinea infection*			
Topical therapy for localized tinea			
	Localized or naïve tinea infection	The ideal treatment duration of topical therapy in patients with localized or naïve tinea infection is 4–6 weeks	76.19%
	Continuation of topical therapy postclinical cure	Topical treatment should be maintained for 2 weeks after visible clinical healing is achieved	83.9%

Systemic therapy for localized tinea			
	Intermittent itraconazole pulse	Intermittent oral itraconazole pulse (200 mg bid for 7 days) is NOT used for localized infection	83.33%

*Recalcitrant tinea*			
	Intermittent itraconazole pulse therapy	Intermittent oral itraconazole pulse (200 mg bid for 7 days each with 3-week interval between 2 pulses) is not used	88.89%

*Others*			
		Medications administered in higher doses over shorter periods are less likely to lead to resistance than those given in lower doses over extended durations	100%

**Table 7:** Therapeutic regimens for antifungal medications.

Drug	Dosing
Itraconazole	The existing treatment protocol involves administering itraconazole at a dose of 200 mg per day for a duration of 4 weeks. For the management of chronic dermatophytosis, a higher dose of itraconazole (200 mg BD) should be used. The recommended itraconazole dosage is 3–5 mg/kg/day for children, while adults are typically prescribed 100 mg once daily for at least 3 weeks [33].

Terbinafine	The current therapeutic regimen includes terbinafine 250 mg daily for 2 weeks.

Griseofulvin	The current dose of griseofulvin is 10–20 mg/kg/day for children for 8 weeks and for adult naïve cases is 500 mg/day for 8 weeks. For chronic/steroid modified tinea/recalcitrant (CH/SMT/RCL) cases, the dose is 750–1000 mg/day for 8 weeks.

Fluconazole	Fluconazole treatment regimens include a daily dose of 3–6 mg/kg for children over 8 weeks. For treatment-naïve adults, the dosage ranges from 50 to 100 mg daily for 4 weeks or 150–300 mg once weekly for 8 weeks. In cases of chronic, steroid-modified, or recalcitrant infections in adults, the recommended regimen is 100 mg daily for 6 weeks or 150 mg three times a week for 8 weeks.

**Table 8:** Consensus on dosing for management of dermatophytosis.

#	Parameter	Consensus	% agreement
*Localized/naive tinea infection*			
	Itraconazole	Itraconazole 100 mg BD	81.82%
	Terbinafine	Terbinafine 250 mg OD	95.24%
	Griseofulvin	Griseofulvin is NOT used for the management of localized/naive tinea infection	76.19%

*Recalcitrant tinea infection*			
	Terbinafine	Terbinafine 250 mg BD	90%

	Topical steroids	Topical steroids are absolutely contraindicated in the management of tinea infection	76.19%

**Table 9:** Consensus on the management of various types of tinea infection.

#	Parameter	Consensus	% agreement
*Tinea pedis*			
	Spread of tinea pedis	Tinea pedis often serves as a reservoir for dermatophyte infections of other anatomical sites	78.95%
	Treatment of recalcitrant lesions	A combination of topical and systemic antifungal agents is used to manage recalcitrant lesions	96.6%

*Tinea cruris*			
	Treatment of recalcitrant lesions	The management of recalcitrant tinea cruris involves the use of both systemic and topical antifungal therapies	100%

*Tinea corporis*			
	Treatment of extensive lesions	For treating extensive lesions, a combination of topical and systemic antifungal therapy is commonly preferred	96.6%
	Treatment of recalcitrant lesions	The preferred approach for treating recalcitrant lesions is a combination of systemic and topical antifungal therapy	100%

*Tinea capitis*			
	First line of therapy	A combination of topical and systemic antifungal treatment is used as the first-line therapy for managing tinea capitis	85.71%
	Choice of systemic antifungal as 1^st^ line	Griseofulvin and itraconazole are the first-line systemic antifungal for tinea capitis.	75.9%
	Duration of therapy	Systemic antifungal is used for 6–8 weeks for the management of tinea capitis	86.36%

*Tinea onychomycosis*			
	Recurrence rate	Onychomycosis is frequently linked to a high rate of recurrence and recalcitrant cases.	90.3%
	Topical antifungal	Topical antifungal monotherapy is used to manage tinea onychomycosis in cases where systemic antifungal treatment is not suitable or contraindicated.	94.12%
	First line	Itraconazole can be administered either continuously at 200 mg per day for 12–16 weeks or as pulse therapy with a dosage of 400 mg per day	77.4%

**Table 10:** Consensus on the supplemental management of tinea infection.

#	Parameter	Consensus	% agreement
	Skin hygiene	Skin hygiene is an essential aspect of the treatment of dermatophytosis	100%
	Antihistamine	Oral antihistaminic agents in management of recalcitrant tinea infections	92.9%
	Emollients/moisturizer	Emollients/moisturizers are used in the management of tinea infections	86.2%
	Topical salicylic acid 6%	Topical salicylic acid 6% is NOT recommended for treating recalcitrant tinea infections	78.95%
	TCIs	TCIs are NOT used in the management of recalcitrant tinea infections	100%
	Oral isotretinoin	Oral isotretinoin is NOT used in the management of recalcitrant tinea infections	95%

Abbreviation: TCI, topical calcineurin inhibitors.

**Table 11:** Percentage and reason for statements that did not reach consensus.

#	Parameter	Consensus	% agreement	Reason for not reaching a consensus
*KOH microscopy*				
	Patient profiles	For research/study purposes	33.33%	In clinical practice, dermatophytosis is diagnosed based on symptoms and clinical evaluation. KOH microscopy is not routinely used because of the added cost; the delay in test reports may delay treatment
		Treatment failure	29.17%	

*Fungal culture*				
	Patient profiles	For research/study purposes	62.50%	Fungal cultures are usually not recommended due to lack of lab facilities
		Refractory infection/treatment failure	58.33%	
		Doubtful cases	41.67%	

*Diagnostics*				
	Sensitivity and specificity of diagnosis depend on	Appropriateness of the sample collection	70.83%	Individual experiences
		Adequacy of the sample	58.33%	
		Personnel expertise	58.33%	

*Management strategies*				
	Selection of an antifungal agent is based on	History of prior exposure to antifungals	41.67%	Choice of antifungal individualized to each patient, based on symptoms, co-morbidities, and even cost (Nepal)
		Pharmacological properties	37.50%	
		Site and extent of the lesion	33.33%	
	Commonly used systemic antifungal in tinea infection	Itraconazole and terbinafine are the most preferred systemic antifungal in the management of tinea infection	70.83%	—

*Systemic therapy for localized tinea*				
Ideal treatment duration of systemic therapy in patients with localized or naïve tinea infection				
	Continuous oral Itraconazole/terbinafine	The ideal treatment duration of continuous oral itraconazole/terbinafine dose is 4–6 weeks	66.67%	—

*Recalcitrant tinea*				
	Topical therapy for recalcitrant tinea	The ideal treatment duration of topical therapy in patients with recalcitrant tinea infection is 6–8 weeks	61.90%	Practical issues to be considered while recommending topical treatment
	Systemic therapy for recalcitrant tinea	The ideal treatment duration of topical therapy in patients with recalcitrant tinea infection is 6–8 weeks	65%	

*Dosing*				
*Localized/naive tinea infection*				
	Fluconazole	Fluconazole 150 mg once weekly	45%	—
		NOT used	45%	—

*Recalcitrant tinea infection*				
	Griseofulvin	NOT used	68.42%	Fluconazole is not a first-line therapy for recalcitrant infection
	Fluconazole	NOT used	63.16%	
	Itraconazole	Itraconazole 200 mg BD	33.33%	

*Stepping up dose of systemic antifungal*				
		The dose of systemic antifungal is increased in nonresponders	54.17%	High risk of hepatotoxicity

*Topical steroids*				
	Clinical use	Topical steroids are helpful initially in some patients with tinea infection	23.81%	Use of topical steroids should be discouraged in tinea infection
	Patient profiles	Topical steroids are used in patients with eczematisation and irritant dermatitis due to some prior topical application	73.68%	Topical steroids should not be prioritized
	Duration	Topical steroids, if needed, are used for up to 1 week to manage tinea infection	70%	Topical steroids are not used alone. They are used in combination with antifungals

*Tinea pedis*				
	Co-infection with tinea pedis	Bacterial co-infection is often associated with tinea pedis	70%	—
	Treatment of localized/ naïve lesions	Combination of systemic and topical antifungal is used for the management of localized/ naïve tinea pedis	70%	

*Tinea cruris*				
	Treatment of localized/ naïve lesions	Combination of Topical and systemic antifungal is used for the management of localized/ naïve tinea cruris	55%	Experts recommended the use of topical therapy in the management of naïve cases of tinea cruris and corporis (localized lesion), while combination therapy is recommended in recalcitrant tinea cruris
		Topical antifungal is preferred for the management of localized/ naïve tinea cruris	45%	

*Tinea corporis*				
	Treatment of localized/ naïve lesions	Topical antifungal is used for the management of localized/ naïve tinea corporis	52.63%	The experts prefer the combination therapy of oral and topical antifungal
		Combination of systemic and topical antifungal is used for the management of localized/ naïve tinea corporis	47.37%	

*Supplemental therapy*				
	Antibacterial	Bacterial superinfection is usually treated with topical antibacterial + topical antifungal	47.06%	—

*Note:* KOH, potassium hydroxide.

## Data Availability

Data sharing is not applicable to this article as no new data were created or analyzed in this study.
