# Supplementary material for: NEXTODERM: Consensus on Dermatophytosis Diagnosis and Management in Nepal
Source: Dermatol Res Pract. 2025 Oct 7;2025:1347872. doi: 10.1155/drp/1347872 (PMC12520815; doi:10.1155/drp/1347872)
Supplement: Supporting Information — Additional supporting information can be found online in the Supporting Information section. [file 1347872.f1.docx]

**Table 1:** Consensus on Epidemiology of Dermatophytosis

| # | **Parameter** | **Consensus** | **% Agreement** |
| --- | --- | --- | --- |
| 1 | Proportion of Male and Female genders affected by Dermatophytosis | 60:40 proportion of Male: Female genders are affected by Dermatophytosis | 91.30% |
| 2 | Age group | Dermatophytosis is commonly seen in 25-35 years of age group | 95.65% |
| 3 | Reasons for increasing prevalence of recalcitrant dermatophytosis | Prior TCS use | 78.26% |

**Table 2:** Consensus on Definitions of Dermatophytosis.

| # | **Parameter** | **Consensus** | **% Agreement** |
| --- | --- | --- | --- |
| Definitions | | | |
|  | Naïve infection | The individual has no prior history of dermatophytosis or its treatment | 96.2% |
|  | Chronic Dermatophytosis | Dermatophytosis is classified as chronic when it persists for over 6 months to a year, with or without recurrence, despite appropriate treatment | 96.2% |
|  | Recurrent Dermatophytosis | A relapse of dermatophytosis within a few weeks (< 6 weeks) of completing treatment is considered a recurrence of the disease | 84.6% |
|  | Relapse | A relapse occurs when lesions of dermatophytosis return after the patient has remained symptom-free for 6 to 8 weeks post successful treatment | 84.6% |
|  | Recalcitrant dermatophytosis | Persistent glabrous tinea typically occurs in scenarios such as chronic, recurrent, corticosteroid-altered, or treatment-resistant cases, where there is minimal or no improvement with standard therapies | 96.2% |

**Table 3:** Consensus on diagnosis of dermatophytosis

| # | **Parameter** | **Consensus** | **% Agreement** |
| --- | --- | --- | --- |
| KOH microscopy | | | |
|  | Clinical recommendation | KOH Microscopy is recommended in patients with Dermatophytosis | 88.5% |
|  | % of cases advised for KOH microscopy | KOH microscopy is recommended in less than 10% of cases | 77.78% |
|  | Patient profiles | Doubtful cases | 79.17% |
| Fungal culture | | | |
|  | % of cases advised for fungal culture | Fungal culture is recommended in less than 10% of dermatophytosis cases | 80% |
| Others advise | | | |
|  | Transportation of specimen | Black paper is used to transport the specimen | 80% |
|  | Use of Sensitivity pattern | Knowing the sensitivity pattern of dermatophytes from chronic/recurrent/recalcitrant Tinea cases will help to develop optimum treatment guidelines | 96.2% |
|  | Dermatoscope | Dermatoscope helps in the management of Dermatophytosis | 95% |
| Other investigations | | | |
|  | LFTs/RFTs before treatment initiation | LFTs/RFTs are **NOT** to be advised at the beginning of systemic antifungal treatment | 85% |
|  | LFTs/RFTs for toxicity monitoring | LFT and RFT should be monitored if any systemic anti-fungal is advised to the patients | 84.6% |

Abbreviations: KOH, Potassium hydroxide; LFTs, Liver function tests; RFTs, Renal function tests.

**Table 4:** Consensus on the management of Tinea infection

| # | **Parameter** | **Consensus** | **% Agreement** |
| --- | --- | --- | --- |
| Management of Tinea infection | | | |
|  | Treatment strategy | Combination therapy is the most widely recommended approach for managing patients with tinea infections | 96.7% |
|  | Commonly used topical antifungal in tinea infection | Luliconazole and Butenafine are the top-choice topical antifungals commonly used in the treatment of tinea infections. | 84.6% |
|  | Duration of topical antifungal | Topical antifungal agents are recommended for another 2 weeks after the lesions of tinea infection have resolved | 93.5% |
|  |  | Topical antifungal agents are recommended to be applied extending 2 cm beyond the edges of the lesion | 93.5% |
|  |  | Use a course of oral antibiotics if secondary infection coexists | 83.9% |
|  | Duration of systemic antifungal | Systemic antifungal agents are recommended for another 2 weeks after clearance of lesions of tinea infection | 80.95% |

**Table 5:** Consensus on combination therapy in the management of recalcitrant tinea infection.

| # | **Parameter** | **Consensus** | **% Agreement** |
| --- | --- | --- | --- |
|  | Type of combination therapy | The use of both systemic and topical antifungal agents in combination is a commonly favored approach for treating tinea infections | 93.5% |
|  | Patient profile | Patients with extensive or persistent tinea infections are often treated with a combination of systemic and topical antifungal medications | 75% |
|  | Most preferred systemic and topical antifungal combination | Oral Itraconazole plus topical azoles | 75% |
|  | Whitfield ointment | Topical antifungal and Whitfield ointment are NOT used for recalcitrant tinea | 76.19% |

**Table 6:** Consensus on duration for management of dermatophytosis

| # | **Parameter** | **Consensus** | **% Agreement** |
| --- | --- | --- | --- |
| **Localized and Naïve tinea infection** | | | |
| Topical therapy for localized tinea | | | |
|  | Localized or naïve tinea infection | The ideal treatment duration of topical therapy in patients with localized or naïve tinea infection is **4-6 weeks** | 76.19% |
|  | Continuation of topical therapy post-clinical cure | Topical treatment should be maintained for **2 weeks after visible clinical healing is achieved** | 83.9% |
| Systemic therapy for localized tinea | | | |
|  | Intermittent Itraconazole pulse | Intermittent oral Itraconazole pulse (200mg bid for 7 days) is **NOT** used for localized infection | 83.33% |
| **Recalcitrant tinea** | | | |
|  | Intermittent Itraconazole pulse therapy | intermittent oral Itraconazole pulse (200mg bid for 7 days each with 3 weeks interval between 2 pulses) is not used | 88.89% |
| Others | | | |
|  |  | Medications administered in higher doses over shorter periods are less likely to lead to resistance than those given in lower doses over extended durations | 100% |

**Table 7:** Therapeutic regimens for antifungal medications

| Drug | Dosing |
| --- | --- |
| Itraconazole | The existing treatment protocol involves administering itraconazole at a dose of 200 mg per day for a duration of four weeks.  For the management of chronic dermatophytosis, a higher dose of Itraconazole (200 mg BD) should be used.  The recommended itraconazole dosage is 3–5 mg/kg/day for children, while adults are typically prescribed 100 mg once daily for at least three weeks.^33^ |
| Terbinafine | The current therapeutic regimen includes Terbinafine 250 mg daily for 2 weeks. |
| Griseofulvin | The current dose of Griseofulvin is 10-20mg/kg/day for children for 8 weeks and for adult Naïve cases is 500 mg/day for 8 weeks.  For chronic/steroid modified tinea/recalcitrant (CH/SMT/RCL) cases the dose is 750-1000 mg/day for 8 weeks. |
| Fluconazole | Fluconazole treatment regimens include a daily dose of 3–6 mg/kg for children over 8 weeks. For treatment-naïve adults, the dosage ranges from 50–100 mg daily for 4 weeks or 150–300 mg once weekly for 8 weeks. In cases of chronic, steroid-modified, or recalcitrant infections in adults, the recommended regimen is 100 mg daily for 6 weeks or 150 mg three times a week for 8 weeks. |

**Table 8:** Consensus on dosing for management of dermatophytosis

| # | **Parameter** | **Consensus** | **% Agreement** |
| --- | --- | --- | --- |
| Localized/naive tinea infection | | | |
|  | Itraconazole | Itraconazole 100mg BD | 81.82% |
|  | Terbinafine | Terbinafine 250mg OD | 95.24% |
|  | Griseofulvin | Griseofulvin is NOT used for the management of localized/naive tinea infection | 76.19% |
| Recalcitrant tinea infection | | | |
|  | Itraconazole | Itraconazole 200mg BD | 33.3% |
|  | Terbinafine | Terbinafine 250mg BD | 90% |
| Stepping up a dose of systemic antifungal | | | |
|  | Topical steroids | | |
|  | Clinical use | Topical steroids are absolutely contraindicated in the management of tinea infection | 76.19% |

**Table 9:** Consensus on management of various types of tinea infection.

| # | **Parameter** | **Consensus** | **% Agreement** |
| --- | --- | --- | --- |
|  | Tinea pedis |  |  |
|  | Spread of tinea pedis | Tinea pedis often serves as a reservoir for dermatophyte infections of other anatomical sites | 78.95% |
|  | Treatment of recalcitrant lesions | A combination of topical and systemic antifungal agents is used to manage recalcitrant lesions | 96.6% |
|  | Tinea Cruris |  |  |
|  | Treatment of recalcitrant lesions | The management of recalcitrant tinea cruris involves the use of both systemic and topical antifungal therapies | 100% |
|  | Tinea Corporis |  |  |
|  | Treatment of extensive lesions | For treating extensive lesions, a combination of topical and systemic antifungal therapy is commonly preferred | 96.6% |
|  | Treatment of recalcitrant lesions | The preferred approach for treating recalcitrant lesions is a combination of systemic and topical antifungal therapy | 100% |
|  | Tinea capitis |  |  |
|  | First line of therapy | A combination of topical and systemic antifungal treatment is used as the first-line therapy for managing tinea capitis | 85.71% |
|  | Choice of systemic antifungal as 1^st^ line | Griseofulvin and Itraconazole are the first line systemic antifungal for tinea capitis. | 75.9% |
|  | Duration of therapy | Systemic antifungal is used for 6-8 weeks for the management of tinea capitis | 86.36% |
|  | Tinea onychomycosis |  |  |
|  | Recurrence rate | Onychomycosis is frequently linked to a high rate of recurrence and recalcitrant cases. | 90.3% |
|  | Topical antifungal | Topical antifungal monotherapy is used to manage tinea onychomycosis in cases where systemic antifungal treatment is not suitable or contraindicated. | 94.12% |
|  | First line | Itraconazole can be administered either continuously at 200 mg per day for 12 to 16 weeks or as pulse therapy with a dosage of 400 mg per day | 77.4% |

**Table 10:** Consensus on management of various types of tinea infection

| # | **Parameter** | **Consensus** | **% Agreement** |
| --- | --- | --- | --- |
|  | Skin hygiene | Skin hygiene is an essential aspect of the treatment of Dermatophytosis | 100% |
|  | Antihistamine | Oral anti-histaminic agents in management of recalcitrant tinea infections | 92.9% |
|  | Emollients/moisturizer | Emollients/moisturizers are **used** in the management of tinea infections | 86.2% |
|  | Topical salicylic acid 6 % | Topical salicylic acid 6% is **NOT** recommended for treating recalcitrant tinea infections | 78.95% |
|  | TCIs | TCIs are **NOT used** in the management of recalcitrant tinea infections | 100% |
|  | Oral isotretinoin | Oral isotretinoin is **NOT used** in the management of recalcitrant tinea infections | 95% |

Abbreviation: TCI, Topical calcineurin inhibitors.
